# Supplementary material for: MechanoProDB: a web-based database for exploring the mechanical properties of proteins
Source: Database (Oxford). 2024 Jun 5;2024:baae047. doi: 10.1093/database/baae047 (PMC11152175; doi:10.1093/database/baae047)
Supplement: baae047_Supp [file baae047_supp.zip › suppl_data/Table S1.docx]

|  | all alpha proteins | all beta proteins | $\alpha+\beta$ proteins | $\alpha/ \beta$ proteins | small proteins |
| --- | --- | --- | --- | --- | --- |
| $N-U$ | 42.8% | 82.5% | 58% | 0 | 100% |
| $N-I-U$ | 14.2% | 12.5% | 33% | 0 | 0 |
| $N-I-I-U$ | 14.2% | 0 | 0 | 100% | 0 |
| $N-I-I-I-U$ | 28.5% | 5% | 0 | 0 | 0 |
| $N-I-I-I-I-U$ | 0 | 0 | 8% | 0 | 0 |
