# Supplementary figures and images for: MechanoProDB: a web-based database for exploring the mechanical properties of proteins
Source: Database (Oxford). 2024 Jun 5;2024:baae047. doi: 10.1093/database/baae047 (PMC11152175; doi:10.1093/database/baae047)

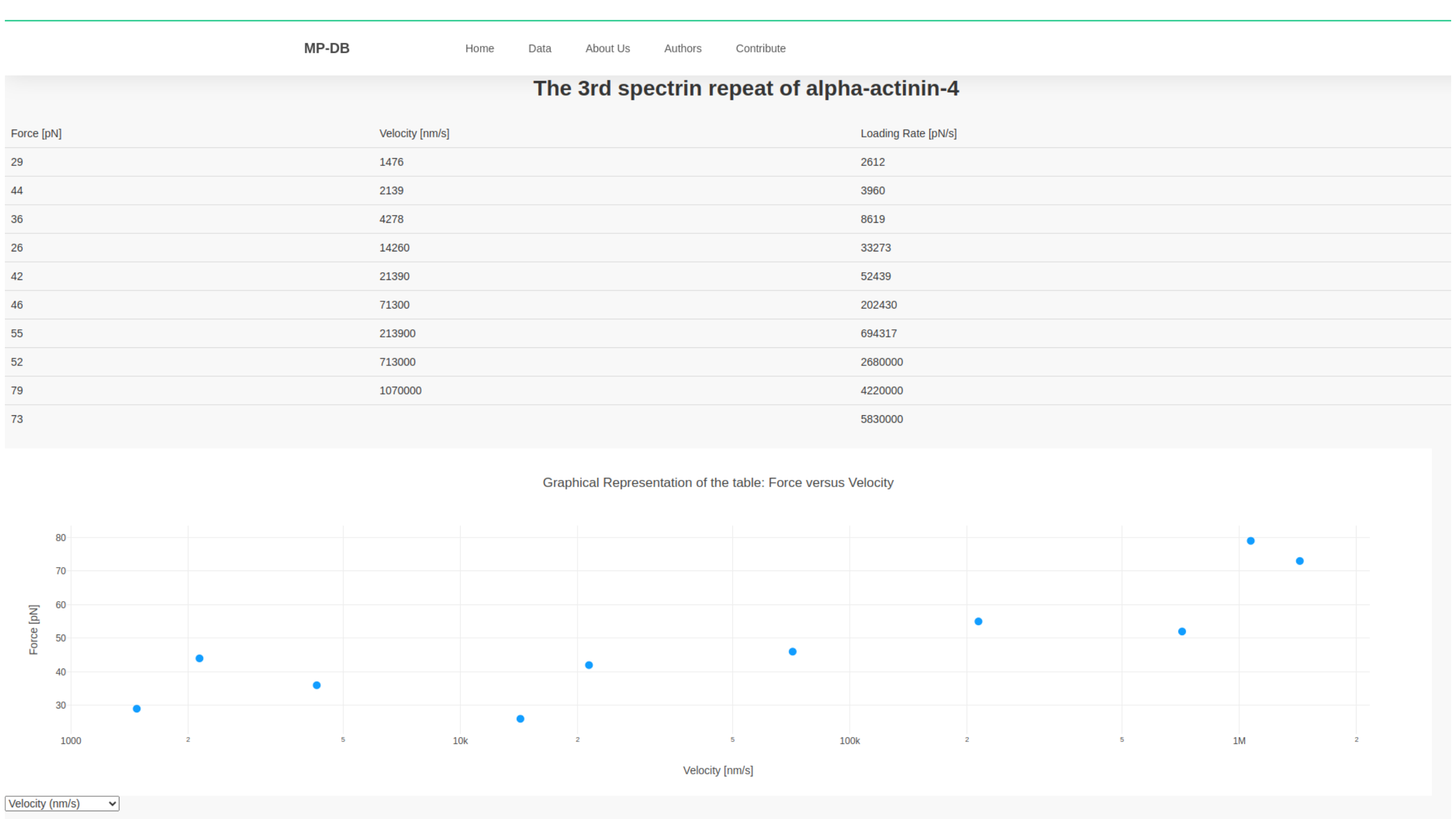

Supplement: baae047_Supp [file baae047_supp.zip › suppl_data/Supp_Figure1_MPDB.tiff]

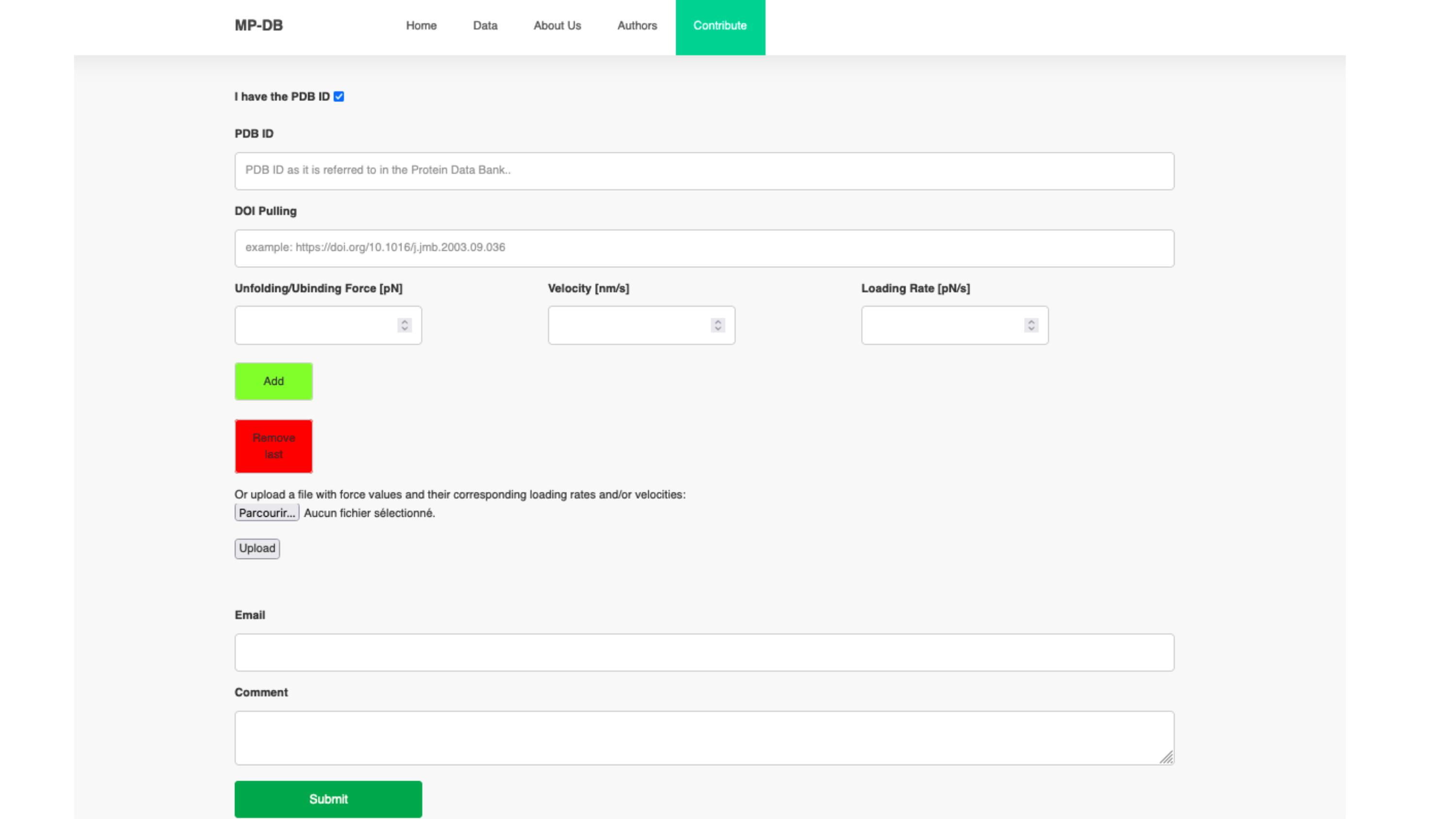

Supplement: baae047_Supp [file baae047_supp.zip › suppl_data/Supp_Figure2_MPDB.tiff]

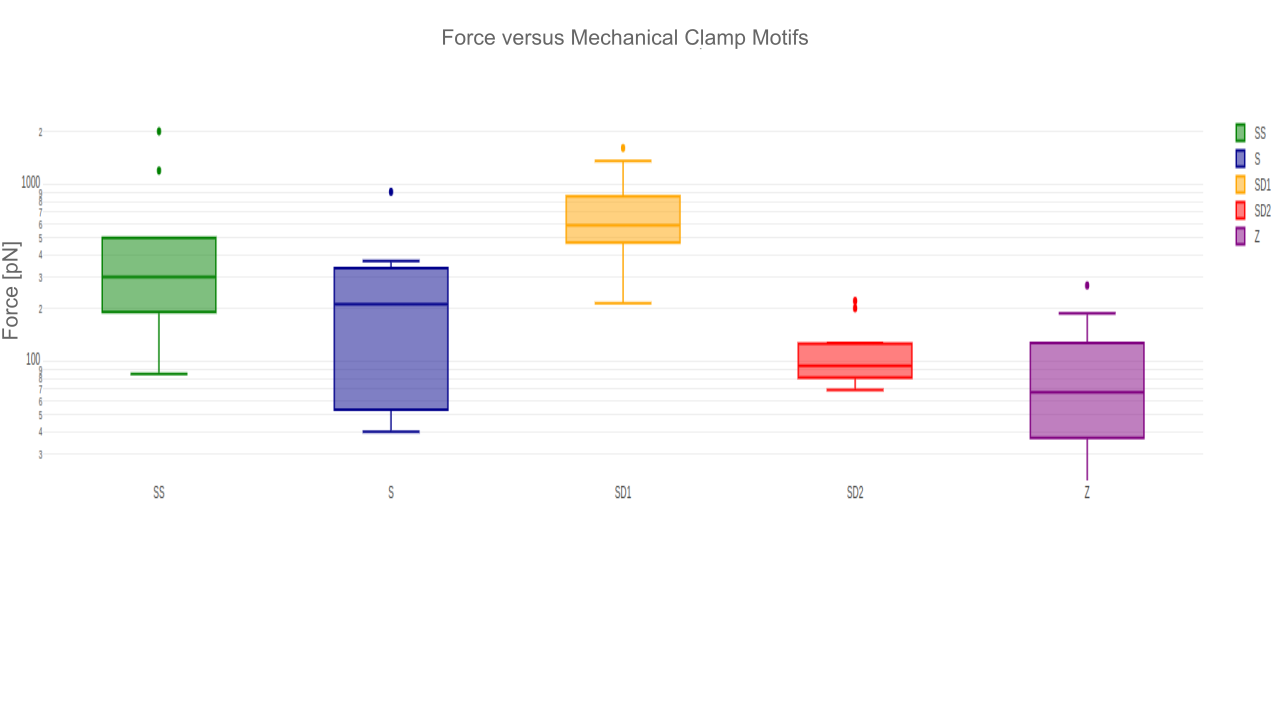

Supplement: baae047_Supp [file baae047_supp.zip › suppl_data/Supp_Figure3_MPDB.tiff]
